# First-Episode Patients Report Cognitive Difficulties in Executive Functioning 1 Year After Initial Episode of Major Depressive Disorder

**DOI:** 10.3389/fpsyt.2021.667238

**Published:** 2021-05-31

**Authors:** Marit Schmid, Åsa Hammar

**Affiliations:** ^1^Department of Welfare and Participation, Faculty of Health and Social Sciences, Western Norway University of Applied Sciences, Bergen, Norway; ^2^Division of Psychiatry, Haukeland University Hospital, University of Bergen, Bergen, Norway; ^3^Department of Biological and Medical Psychology, University of Bergen, Bergen, Norway

**Keywords:** depression, first episode, cognitive functioning, residual cognitive symptoms, behavior rating inventory of executive function

## Abstract

**Objective:** Major Depressive Disorder (MDD) is associated with cognitive impairment in general, and Executive Functioning (EF) in particular, even in remitted phase of the disorder, suggesting residual cognitive symptoms. The aim of the present study was to investigate self-reported EF 1 year after the first episode of MDD and to explore this in relation to depressive mood symptoms, remission and relapse.

**Method:** The study included data from 24 patients and 23 healthy control subjects 1 year after the patients' initial first episode of MDD. All participants completed the Behavior Rating Inventory of Executive Functioning-Adult version (BRIEF-A), a standardized self-report measure of perceived EF in everyday life, measuring nine different EF. Total index scores for metacognitive functions, behavior/emotional regulation functions and a global EF score is also calculated.

**Results:** The patient group in total, independent of symptom status, reported significantly lower EF in all indexes compared to the healthy controls 1 year after the initial episode. However, higher depressive mood symptom load correlated with self-reported difficulties in metacognitive functions and poor global EF scores. Regulatory control of behavior and emotional responses did not show such strong association with mood symptoms, but low self-report scores on this measure was associated with relapse during the first year after the initial episode.

**Conclusion:** First-episode patients report significant lower executive functioning in everyday life compared to individually matched healthy controls, 1 year after onset, independent of symptom load. Residual cognitive symptoms seem to be evident and associated with risk of relapse and should be targeted in treatment and prevention of recurrence in MDD.

## Introduction

Major depressive disorder (MDD) is the most prevalent mental disorder (1), and is characterized by significant disability, including reduced social and work ability, and poor quality of life (2). An important challenge is the relapsing and recurrent nature of MDD (3), and despite relatively high-quality medical and therapeutic treatment options where most patients achieve symptom relief and remission, a high proportion of MDD patients suffer from a relapse of depressive symptoms and recurrence of depressive episodes (4). Studies have found that ~50% of patients experience a relapse within the first 2 years after the initial episode (5). Furthermore, the vulnerability and risk of relapse is enhanced for each episode experienced (6). The factors contributing to the high relapse risk and recurrent course in MDD are not fully understood. Although residual depressive mood symptomatology is recognized to be a significant factor contributing to general disability, relapse and recurrence in MDD (7), longitudinal studies of MDD patients clearly show that mood symptoms alone are unlikely to present a sufficient target for interventions (8). Studies have shown that neurocognitive functioning might be a key determinant affecting this relationship (9–11) with authors stressing the importance of recognizing cognitive impairments in clinical practice to improve clinical interventions (12). Importantly, studies find that disability and poorer quality of life are found to persist for years despite symptom reduction and remission (13, 14), and cognitive impairment has been found to be a significant illness characteristic mediating the association between MDD and disability in everyday life (15, 16). One study found that cognitive impairment in first episode patients, especially Executive Functioning (EF) impairment, predicted symptom severity in follow-up, highlighting the importance of interventions and treatment of cognitive deficits at an early stage of illness (11). Neurocognitive impairment in first episode patients has also been associated with risk of relapse (5).

Research over the past two decades has documented neurocognitive impairment to be a core feature of MDD and characteristic of patient groups across the symptomatic phases (10, 17–19). Findings have revealed impairment in several cognitive domains such as processing speed, memory, attention and EF (18). Importantly, although cognitive improvement parallel to symptom recovery from the depressive episode has been reported (20), several studies find cognitive impairment, and especially EF impairment, to persist despite symptom reduction and remission (9, 18, 21, 22). Longitudinal studies following patients from the acute phase of illness *through* phases of symptom reduction and remission have found that impairment in psychomotor speed and memory often normalize more in step with symptom reduction and remission, while impairment of attention and especially EF persist beyond remission, representing a prolonged neurocognitive impairment despite symptom relief (22). Importantly, this pattern of prolonged EF impairment has also been found in longitudinal studies following first episode MDD patients (5, 23), showing that these deficits are already present early in the course of the illness and independent of depressive illness course. It has been suggested that prolonged EF impairments represent stable traits in MDD, which are possibly present before the first depressive episode is even experienced (24). Thus, EF is recognized as an important cognitive function to target in MDD. However, EF is a broad, multi-functional concept, making it challenging to investigate. EF is commonly defined as a set of cognitive control processes that consist of several functions important for governing behavior, including emotional regulation, the ability to perform complex activities such as planning, organizing, and the ability to sustain attention and self-management (25, 26). When studying EF separately, a majority of studies find the EF functions of inhibition and mental flexibility to be of importance in MDD. In particular, the function of inhibition has been recognized as a core EF function (27, 28), representing a function that is common to most EF functions, such as mental flexibility, working memory, planning and problem solving, initiating and emotional regulation. Poor EF in general, and poor inhibition in particular, has been associated with lower treatment response (29), and to represent a risk of relapse (5). Furthermore, poor EF has been associated with poor rumination (30, 31) and poor emotional regulation (32, 33).

Thus, when poor EF persists during phases of symptom reduction and remission it represents an important residual symptom affecting an individual's recovery following depressive episodes (5, 8, 34). EF is therefore suggested to be a core target in the development of new treatment strategies for MDD (35, 36).

Research on neurocognitive functions in MDD is, however, mostly based on studies using objective neuropsychological tests or experimental designs. There are few empirical studies investigating MDD patients' subjective experience of cognitive difficulties, and how this affects everyday life. Knowledge of this is important due to the low correlation between objective and subjective measures (37, 38) and the indication of low ecological validity of objective test measures (39). Objective neuropsychological tests in general have been found to have low ecological validity in predicting everyday functioning (40). The lack of ecological validity is especially prominent in measures of EF performance (39, 41). Toplak et al. (39) argue that objective test measurements only reveal few or specific EF impairments; this is in contrast to self-reported measures, which expose more severe and general impairments in everyday life. Furthermore, subjective reported cognitive impairments in MDD have been found to correlate more with socio-occupational functioning than objective ratings (37). Thus, subjective reports and descriptions may aid clinicians to better understand the impact neurocognitive dysfunctions have in everyday life, and to target more precise treatment interventions.

Studies addressing self-reported cognitive impairment in general (using interviews, questionnaires and check lists) have yielded important knowledge showing cognitive impairment both during phases of depression and remission (7, 17, 37, 38). One qualitative study found neurocognitive symptoms, such as inability to concentrate, poor EF, memory, planning and problem solving, and poor organization to be frequently reported by patients with MDD and were described by patients to largely affect their everyday functioning leading to low coping and self-esteem (42). A matter of importance when investigating subjective self-reporting of cognition in MDD is to control for the impact of depressive mood symptoms. Severity of illness, illness chronicity, and younger age have been found to be positively correlated with higher self-reported impairments (17), with one study also showing that subjectively experienced cognitive impairment was predicted by depression severity (38). Specific for the assessment of self-reported EF using BRIEF-A, one study reported a positive correlation between severity in symptom load and self-reported impairment across diagnostic groups (43). Thus, when investigating self-reported cognitive function, one should correlate these reports to severity of depressive mood symptoms.

Another important issue when investigating self-reported EF impairment is the use of rating scales. Studies are divergent with regard to the subjective ratings used. Some studies have used traditional symptom scales or psychiatric interviews tapping questions with cognitive content (7) while others have used more detailed questionnaires tapping several cognitive functions (38), which might not be specific enough to target how EF impairments affect behavior in everyday life. This emphasizes the importance of more systematically assessing self-report questionnaires that specifically target EF, in order to enhance the ecological validity of EF impairment. There is also a lack of studies that have systematically investigated self-reported EF impairment in MDD in a standardized manner.

The aim of the present study was therefore to investigate self-reported EF in MDD 1 year after the initial episode of MDD and to explore this in relation to mood symptoms, remission and relapse. The standardized questionnaire developed to assess EF in everyday life, The Behavior Rating Inventory of Executive Function-Adult Version (BRIEF-A) [(44); Norwegian translation (45)] was administered. The present study is part of a comprehensive longitudinal project following a group of first episode MDD patients for 1 year, investigating EF using objective neuropsychological tests, and measuring patients in both the acute phase of illness and after 1 year when the patient group was in remission. Results revealed persistent EF impairment in objective EF tests despite symptom reduction and remission [for detailed descriptions see (5, 46)]. In the 1 year follow-up assessment, the study included subjective self-report measures to enhance our understanding of EF following initial episode.

Objectives and research questions

1. Do first-episode MDD patients report significantly lower EF compared to healthy controls 1 year after the initial episode?
2. Are self-reported difficulties in EF associated with depressive symptom load 1 year after the initial episode?
3. Are self-reported difficulties in EF related to relapse during the first year after the initial episode?

Based on the previous literature, we expected the patient group, independent of symptom load or remission, to report significantly lower EF compared to the control group. Furthermore, for the patient group, we expected that poorer EF functions would be associated with symptom load and relapse during the first year after initial episode.

## Materials and Methods

### Study Subjects and Inclusion/Exclusion Criteria

The study initially (T1) included 30 patients (16 males and 14 females) meeting the DSM-IV criteria for a unipolar first episode MDD diagnosis using the MINI-International Psychiatric Structural Interview (47). To assess the severity of depression at both T1 and at the 1 year follow-up (T2), the structural rating scale Montgomery Åsberg Depression Rating Scale (MADRS) (48) was administered.

At T1, prospective patients were identified through cooperation with doctors and psychologists in primary healthcare, and the study coordinators were contacted if patients met the inclusion criteria and agreed to be contacted by the study coordinators for inclusion. Inclusion criteria for the patient group at T1 were a diagnosis of first episode MDD and a minimum score of 20 on the MADRS (indicating moderate to severe depression). Patients diagnosed with, or receiving treatment for, depression earlier in life; patients with known brain injury, severe somatic disorders, or alcohol or substance abuse; and patients who had been treated with electroconvulsive therapy or who were psychotic or who had experienced psychosis earlier in life, were excluded from the study.

At T1, a control group of 30 subjects was included who were individually matched to the patient group with respect to age, gender, and years of education (within a ± 2 year range). Exclusion criteria for the control group were a history of brain damage, any severe somatic disorder, any mental disorder, and alcohol or substance abuse. Regarding the control group, all participants were asked if they had a history of psychiatric symptoms or diagnosis, and they were asked about depressive symptoms at inclusion. To control for the effect of general intellectual abilities (IQ), the two-subtest form of the Wechsler's Abbreviated Scales of Intelligence (WASI) (49) was administrated at T1.

At both inclusion (T1) and the 1 year follow-up (T2), all subjects were assessed according to demographic variables and with a standardized neuropsychological test [for a more detailed description, see (5, 46)]. All subjects were asked to participate in the follow-up assessment 1 year later. One year follow-up data from two patients were missing due to drop out. The study coordinators were unable to contact one of these patients, and the other did not want to participate in the follow-up assessment.

### Dividing the Patient Group According to Being in Remission and According to Having had a Relapse Since Initial Episode and the Use of Antidepressant Medication

At T2, patients were evaluated according to being in remission and if they had experienced a relapse since inclusion. The definition of a remission and relapse was based on suggested operational criteria for outcomes in depression designed by Frank et al. (50) and Rush et al. (51), and remission was also defined according to suggested cut-off scores on MADRS (48) provided by Hawley et al. (52) and Rush et al. (51). At T2, patients were divided according to depression severity into a Remitted Group (RG) (*N* = 13) with MADRS score ≤ 9 and a Depressed Symptom Group (DSG) (*N* = 11) with MADRS score > 9 (which indicates residual depressive symptoms). Furthermore, in order to detect whether patients had experienced a relapse of their depressive illness, all patients were interviewed retrospectively as regards the course of their symptoms since inclusion and during follow-up at T2. A drawn timeline from inclusion to follow-up was used during the interview to obtain the most accurate recall possible of the previous year's events. A relapse was defined as a return to a fully symptomatic state of depression after a minimum 3 week period during which minimal symptom status is maintained (remission). To fulfill the criteria of a relapse, the subject had to report the relapse period as having lasted a minimum of 2 weeks. In the present project a relapse was further defined as a period during which the subject reported difficulties performing at an optimal level in areas such as school, work or social functioning. All patients were interviewed by a psychologist. At T2, this categorization of patients resulted in three different groups, where 11 patients had experienced a relapse during the first year since the initial episode (Relapse Group), 10 patients had not experienced a relapse (No Relapse Group), and the remaining 3 patients had no change in symptom load since inclusion. Patients were also divided according to the use of antidepressant medication (*N* = 13) or not (*N* = 11) to explore if this would influence the results.

BRIEF-A was administrated at T2, 1 year after inclusion. Three patients and five control subjects did not complete or deliver the BRIEF-A for various reasons. One patient was identified as an outlier by the statistical programme due to extreme and invalid BRIEF-A scores, and data from this patient were removed from the analysis. Thus, BRIEF-A data from 24 patients and 23 control subjects were ultimately included, and clinical and demographic data for the two groups are shown in Table 1.

**Table 1:** Clinical and demographic variables for the patient and control groups at T2.

	**Patients (*****n*** **=** **24)**		**Controls (*****n*** **=** **23)**			
**Variable**	**M**	**SD**	**M**	**SD**	***t***	***p***
Age	27.38	5.63	27.43	5.09	−0.038	0.97^a^
Male/Female	13/11	NA	13/10	NA	NA	NA
Education (years)	14.25	1.75	15.00	1.78	−1.46	0.153^a^
Total IQ (WASI)	119.04	7.26	120.87	8.79	−0.779	0.440^a^
MADRS T1	24.83	3.94	^**^	^**^	NA	NA
MADRS T2	10.25	6.38	^**^	^**^	NA	NA

** *MADRS score is not reported for healthy controls. NA, not applicable*.

a *Independent samples t-test*.

*MADRS, Montgomery and Åsberg Depression Rating Scale; WASI: Wechsler Abbreviated Scale of Intelligence*.

### Procedure

Informed consent was obtained from all participants, and the study was performed in accordance with the Helsinki Declaration of the World Medical Association Assembly. The Regional Committee for Medical Research Ethics and The Norwegian Data Inspectorate approved the study.

### Behavior Rating Inventory of Executive Function-Adult Version (BRIEF-A)

Perceived EF in everyday life was measured using the BRIEF-A self-report (44). The Norwegian translated version was used (45). The BRIEF-A is a standardized self-report measure of executive function in everyday life for adults aged 18 to 90 years. It consists of 75 items reflecting behaviors to be rated as *often a problem (score 3), sometimes a problem (score 2)*, or *never a problem (score 1)* in everyday life during the last months. Two summary indexes are calculated—the Metacognition Index (MI) and the Behavior Regulation Index (BRI). These summary indexes consist of different EFs (44). The MI is composed of the five clinical scales Initiate, Working memory, Plan/organize, Task monitor and Organization of Materials, and the BRI is composed of the four clinical scales Inhibit, Shift, Emotional Control and Self-Monitor. A Global Executive Composite (GEC) is calculated based on all clinical EF scales (MI + BRI) to give a total EF score. Raw scores are calculated for each clinical scale and for the summary indexes (MI and BRI) and total score (GEC). The present study used raw scores for the nine different scales and for the total MI, BRI, and GEC scores. Raw scores are used in the main analysis because no Norwegian norms for BRIEF-A are available. However, T-scores (mean and standard deviations) are added in **Table 4** for clinical relevance. Studies that have included Norwegian norm-samples have shown that this is a feasible and valid instrument for use in Norway. However, these studies have also shown that the clinical cut-off needs to be adjusted when interpreting the result because the Norwegian norm-samples score ½ to 1 SD below the original American norms (43, 53). The BRIEF-A also includes the three validity scales of Negativity, Infrequency, and Inconsistency. All BRIEF-A reports were evaluated with regard to validity in accordance with the manual.

### Data Scoring and Analyses

Independent sample *t*-tests were computed to compare the patient and control group, and the two patient groups, as regards age, years of education and IQ scores (see Tables 1, 2). A one-way between-groups multivariate analysis of variance was performed to compare the patient and control group on the nine different BRIEF-A subscale raw scores and on the MI and BRI indexes and the GEC. The dependent variables used were BRIEF-A raw scores, and the independent variable was group (patient or control). Higher scores reflected greater self-reported impairment in EF. The relationship between BRIEF-A score and severity of depression symptoms (measured by MADRS at T1 and T2) were examined for the patient group by Pearson's correlation coefficient. To separate patients in remission from patients with residual depressive symptoms, the patients were divided according to depression severity measured with MADRS into a Remitted Group (RG) and a group with residual depressive symptoms, the Depressed Symptom Group (DSG). These groups were compared to the Control Group (CG) on the main BRIEF-A indexes using a between-groups multivariate analysis of variance with *post hoc* analysis. Mean and Standard deviations are provided for scores adjusted to American norms (T-score) for clinical practical purposes. The patient group were also divided according to the experience of relapse during the year since initial episode using an independent samples *T*-test. An independent samples *T*-test was also carried out to explore the difference between patients that used medication and the patients that did not regarding symptom severity (MADRS) and the EF indexes. Preliminary assumption testing was conducted to check for any statistical violations, and the data were examined for univariate and multivariate outliers. One patient was identified as an outlier by the statistical program due to extremely high scores, and data from this patient were removed. When analyzing the data, the assumption of homogeneity of variance was not met for the one-way between-groups multivariate analysis of variance and the *post hoc* analysis, thus the alpha level was interpreted with caution and set to *p* < = 0.001.

**Table 2:** Clinical and demographic variables for the Remitted Group (RG) (MADRS ≤ 9) and the Depressed Symptom Group (DSG) (MADRS > 9).

	**RG, MADRS** **≤** **9 (*****N*** **=** **13)**		**DSG, MADRS** **>** **9 (*****N*** **=** **11)**			
**Variable**	**M**	**SD**	**M**	**SD**	***t***	***p*^a^**
Age (years)	27.15	6.07	27.64	5.33	0.205	0.483
Male/Female	8/5	NA	5/6	NA	NA	NA
Education (years)	14.85	1.28	13.55	2.02	−1.92	0.068
MADRS^*^	5.46	2.18	15.91	4.74	6.72	<0.001
Total IQ (WASI)	118.54	8.24	119.64	6.23	0.362	0.721

a *Independent samples t-test*.

* *MADRS: equal variances not assumed, statistics reported*.

*MADRS, Montgomery and Åsberg Depression Rating Scale; WASI, Wechsler Abbreviated Scale of Intelligence, NA, not applicable*.

## Results

The patient and control groups did not differ with regard to total IQ, age or years of education. There was a relatively high standard deviation for the MADRS score, in which raw scores ranged from 2 to 24 (see Table 1).

When dividing the groups by depression severity according to MADRS, 13 patients had a score ≤ 9 and thus were in remission, the Remitted Group (RG), and 11 patients had a score > 9 indicating the presence residual mood symptoms or mild to moderate depression, the Depressed Symptom Group (DSG). The groups did not differ significantly with regard to total IQ, age, or years of education (see Table 2).

### BRIEF-A

There was a statistically significant difference between the patient group and the control group on all clinical scales and combined indexes on BRIEF-A. The main effect of group on the combined dependent variables was statistically significant [*F*_(9.37)_ = 9.09, *p* < 0.001, Wilks's lambda = 0.31, partial eta squared = 0.69]. When the clinical scales were analyzed separately, the patient group reported significantly greater EF difficulties on all scales (see Table 3 for raw scores, T-scores and statistics).

**Table 3:** BRIEF-A self-report for patient and control groups.

	**Patient group**, ***N*** **=** **24**		**Control group**, ***N*** **=** **23**		**Statistics**		
**BRIEF-A**	**M**	**SD**	**M**	**SD**	**F**	**Sig**.	**Eta Sq**.
Inhibit	11.75	3.05	9.3	1.36	12.38	*p* < 0.001	0.22
Shift	9.83	2.79	6.78	1.17	23.51	*p* < 0.001	0.34
Emotional control	16.33	5.21	11.74	2.91	13.78	*p* < 0.001	0.23
Self-Monitor	7.87	1.82	6.48	0.79	11.41	*p* = 0.002	0.2
Beha.reg (BRI)	45.79	9.89	34.3	4.78	25.32	*p* < 0.001	0.36
Initiate	16.58	4.33	9.82	1.83	47.77	*p* < 0.001	0.52
Work memory	14.54	3.78	8.83	1.27	47.53	*p* < 0.001	0.51
Plan/Organize	18.13	4.56	11	1.2	52.54	*p* < 0.001	0.54
Task monitor	10.88	2.31	6.82	1.15	57.03	*p* < 0.001	0.56
Org. of materials	13.71	4.16	10.13	2.75	11.96	*p* = 0.001	0.21
Meta cog. (MI)	73.83	17.18	46.6	6.32	51.04	*p* < 0.001	0.53
EF Comp. (GEC)	119.63	24.27	80.91	10.12	50.11	*p* < 0.001	0.52

*Mean (M), standard deviation (SD) and statistics (F, Sig., Eta Sq)*.

*Analyses were carried out using raw scores*.

### The BRIEF-A and Depression Severity

There were strong positive correlations between severity of depression at T1 (MADRS) and self-reported EF impairment (BRIEF-A) on the combined index MI (r = 0.498, *N* = 24, *p* = 0.013), the BRI index (r = 0.530, *N* = 24, *p* = 0.008), and GEC (r = 0.569, *N* = 24, *p* = 0.004), with higher severity of depression at inclusion was associated with higher self-perceived difficulties on all EF indexes. There were further a strong positive correlations between severity of depression at T2 (MADRS) and self-reported EF impairment (BRIEF-A) on the combined index MI (r = 0.61, *N* = 24, *p* = 0.002) and GEC (r = 0.59, *N* = 24, *p* = 0.003), with higher severity of depression associated with higher self-perceived difficulties on these EF indexes. There were no significant correlations between depression severity and the BRI index (r = 0.39, *N* = 24, *p* = 0.061). This indicates that depression severity is strongly associated with higher self-perceived impairment in metacognitive functions, but that the association between depression severity and self-reported regulatory control of behavior and emotional responses is not that strong 1 year after initial episode.

### Dividing the Patient Group by MADRS Score

Dividing the patient group according to MADRS score revealed significant differences between the three groups in self-reported EF impairment, with patients in the Remitted Group (RG) (mean MADRS score = 5.46, SD = 2.18, *N* = 13) and Depressed Symptom Group (DSG) (mean MADRS score = 15.91, SD = 4.74, *N* = 11) self-reporting significantly greater impairment in all EF in everyday life on the three main indexes (BRI Index, MI index, GEC Index) compared to the Control Group (CG). The results showed a statistically significant main effect of group on the combined dependent variables [*F*_(4, 86)_ = 15.67, *p* < 0.001, Wilks's lambda = 0.335, partial eta squared = 0.422]. *Post hoc* comparisons using the Tukey HSD test showed that the control group self-reported significantly lower EF difficulties in everyday life compared to the two patient groups on all main indexes. Furthermore, *post hoc* tests also revealed that the DSG self-reported significantly greater EF difficulties compared to the RG group on all the main indexes.

Although the assumption of homogeneity of variance was not met for these analyses, significance levels of <0.001 or 0.013 were reached after correcting for unequal variances across groups (See Table 4 for raw scores and statistics).

**Table 4:** Self-reported EF impairment for the Depressed Symptom Group (DSG); MADRS^*^ > 9, Remitted Group (RG); MADRS^*^ ≤ 9) and the Control Group (CG); MADRS^*^ N/A.

	**DSG, *N* = 11**		**RG, *N* = 13**		**CG, *N* = 23**		**Stat**.	
**BRIEF-A raw scores**	**M**	**SD**	**M**	**SD**	**M**	**SD**	**sig**.	***post hoc***
Beha.reg (BRI)	50.55	10.41	41.77	7.69	34.30	4.78	^**^	DSG < RG < CG
Metacog. (MI)	83.73	13.02	65.46	16.09	46.61	6.33	^*^	DSG < RG < CG
EFComp. (GEC)	134.27	19.31	107.23	21.34	80.91	10.12	^*^	DSG < RG < CG
**BRIEF-A T-scores**	**T**	**SD**	**T**	**SD**	**T**	**SD**		
Beha.reg (BRI)	56.0	10.61	47.08	8.14	39.52	4.69		DSG < RG < CG
Metacog. (MI)	68.64	10.22	54.78	12.22	40.60	4.47		DSG < RG < CG
EFComp. (GEC)	64.45	9.33	51.62	10.15	39.21	4.53		DSG < RG < CG

*Mean (M), standard deviation (SD), and statistics (Sig., post-hoc) for raw scores*.

*T- scores (M) and (SD) are provided.

* *sig < 0.001*.

** *sig = 0.013*.

*T- scores based on American norms (44).

* *MADRS, Montgomery Aasberg Depresssion Rating Scale*.

### The Association Between Relapse During the First Year After the Initial Episode and Self-Reported EF

Dividing the patient group as regards experience of relapse during the 1 year follow-up period revealed that the patients who had experienced a relapse (*N* = 11, M = 9.09, SD = 5.18) did not differ with regard to MADRS score at T2 compared to the patients that did not have a relapse (*N* = 10, M = 8.60, SD = 5.82) [*t*_(19)_ = 0.205, *p* = 0.840]. However, the relapse group (*N* = 11, M = 48.09, SD = 9.91) self-reported significantly more impairment compared to the patients with no relapse experience (*N* = 10, M = 40.40, SD = 5.15) on the BRI scale [*t*_(15, 31)_ = 2.26, *p* = 0.039]. The magnitude of the difference in the means (mean difference = 7.69, 95% CI: 0.449 to 14.93) was large (Eta squared = 0.21). For MI and total GEC, there were no significant differences between the relapse group (MI: M = 72.0, SD = 16.79, GEC: M = 120.09, SD = 25.31) and the no-relapse group (MI: M = 71.40, SD = 18.03, GEC: M = 111.80, SD = 19.40).

### Dividing the Patient Group Concerning the Use of Antidepressant Medication

Dividing the patient group according to those using antidepressant medication (*N* = 13) and those that did not (*N* = 11) showed that there was no difference in clinical measures and self-reported EF difficulties. For the MADRS score at T1 there were no difference between the ones that used antidepressant medication (M = 25.38, SD = 4.53) and the ones that did not (M = 24.18, SD = 3.19) [*t*_(24)_ = 0.737, *p* = 0.469]. For MADRS score at T2 there were no difference between the group that used medication (M = 11.23, SD = 6.53) and those that did not (M = 9.09, SD = 6.27) [*t*_(24)_ = 0.814, *p* = 0.424]. In the BRI Index there were no difference between patients that used medication (M = 48.62, SD = 10.97) and the group that did not (M = 42.45, SD = 10.97) [*t*_(24)_ = 1.567, *p* = 0.131]. On the MI index there were no difference between the group that used medication (M = 76.31, SD = 19.12) and the patients that did not (M = 70.91, SD = 14.94) [*t*_(24)_ = 0.760, *p* = 0.456]. On the GEC index there were no difference between the patients that used medication (M = 124.92, SD = 27.13) and the patients that did not (M = 13.36, SD = 19.80) [*t*_(24)_ = 1.172, *p* = 0.254].

## Discussion

The main aim of the present study was to investigate self-reported EF 1 year after the first episode of MDD in relation to depressive mood symptoms, remission and relapse. In addition, the impact of clinical factors such as the use of medication was explored. In order to broadly assess self-reported EF in everyday life, an extensive and standardized questionnaire, BRIEF-A, was used. Even though BRIEF-A is used frequently in clinical settings to complement objective neuropsychological assessments (41), to our knowledge there are only two studies using BRIEF- A in MDD (54, 55). The former used BRIEF-A to assess effect of cognitive training on patients and the latter was a pilot study with no matched control group.

The results showed that 1 year after their initial episode, first episode MDD patients reported significantly poorer EF in everyday life compared to the healthy control group. Furthermore, the present study found that depression severity measured at inclusion, when patients were in the symptomatic phase, and measured at 1 year after, were associated with self- reported EF abilities at 1 year after initial episode. Symptom load at initial episode was strongly associated with all EF index measures, which may indicate that those patients with higher depressive symptom load at inclusion are the ones that experience most difficulties in EF 1 year later. However, 1 year after initial episode, these associations were only evident for some EF abilities. Depressive mood symptoms correlated strongly with reported EF difficulties in scales measuring metacognitive EF (MI), such as Initiate, Plan/organize, Working memory and Task monitor. There were no such strong association between mood symptoms and self-reported difficulties on scales measuring regulatory control of behavior and emotional responses (BRI), such as Inhibition, Shift (mental flexibility), Emotional Control and Self-Monitor. In order to further explore the impact of depressive mood symptoms on self-experienced difficulties, the patient group was divided according to being in remission (defined as MADRS ≤ 9) or not. Independent of group affiliation, both patient groups reported significantly poorer EF compared to the control group. This shows that difficulties in some EF abilities are still evident despite remission. Moreover, the results showed that patients who had experienced a relapse during the first initial year reported significantly poorer EF in behavioral and emotional regulatory (BRI) functions compared to the patients who did not relapse, indicating that patients that have experienced relapse report more difficulties in these EF abilities 1 year after initial episode The use of antidepressant medication did not have any impact on depressive symptom load at inclusion or 1 year after initial episode or the self-reported EF indexes.

These results support previous findings of self-reported cognition, or EF, being sensitive for depressive symptom load (17, 38, 43) but also support the hypothesis that EF impairment may be independent of symptom severity, representing more stable enduring residual cognitive symptoms affecting the individual ability to regain former daily life functioning (5, 10, 18, 22). These results may indicate that some EF difficulties, and especially functions of inhibition, mental flexibility, emotional regulation and self-monitor may be more stable traits making the individual more vulnerable to relapse. However, the direction of this relationship needs to be further investigated because these patients might experience greater impairments because of their relapse experience, making them more sensitive to own emotional regulation.

The present study further finds a general poorer self-report across several EF. This is in contrast to objective measures which often finds specific EF to be impaired. This may also be the reason that objective and subjective measures seldom correlate. As argued by Toplak et al. (39), subjective reports are more general, in that the patients often report a more general and non-specific impairment which affects them in most daily life activities. Toplak et al. (39) claim that objective measures may address cognition at the core level of neuropsychological functioning, while subjective measures of EF may target situations of higher complexity, demanding more general EF to solve complex behaviors in everyday life. Using subjective ratings may add to our knowledge of how impairment is experienced in everyday life. However, the present results also to some extent mirror previous findings from objective test measures with the indication that some EF, such as the functions of inhibition, emotional regulation, mental flexibility (shift), and self-monitor, may be of particular importance in MDD, representing more stable impairments, less dependent on depressive symptom severity, that possibly heighten the risk for relapse. More specifically, the core EF functions of inhibition and mental flexibility have been found to be important functions for emotional regulation and regulatory control over behavior (30–33). The finding that subjective measures also to some degree identify these impairments to be more stable and independent of symptom severity, adds support to previous studies arguing for the importance of these residual symptoms when understanding the illness course in MMD and targets for treatment.

### Strengths and Limitations

A clear limitation in the present study is the small number of participants and thereby low statistical power. Thus, generalization of the results should be made with caution and results need to be replicated in larger samples. Nevertheless, the significant results between groups despite low N should be considered as robust and valid due to the minor risk of type I error. Moreover, the patients represent a relatively homogenous group, with higher IQ and years of education than average, which also limits generalization and should be borne in mind when interpreting the results. A strength is however that the patient group was well-defined with regard to diagnostic criteria, as well as symptom load, and the control group was matched for gender, age and education. Further, the risk of conduction type II errors has to be considered regarding border significant results.

Another limitation is that the BRIEF-A assessment was not administrated at inclusion when patients were in the acute phase of their illness; this could have added more information. For example, it would have been possible to assess whether the patient group's self-reported EF changed during the course of illness. Further, such data might have more precisely revealed the direction of the relationship between self-reported impairment and relapse, and knowing this we might have been able to identify the patients who are more vulnerable to relapse following their initial episode. Further, although the present study finds that patients in remission report difficulties in EF in everyday life, one cannot exclude reporting bias due to depressive residual symptoms. This is further limited due to the fact the control group was only asked about symptoms and not screened for depressive symptoms. By adding objective test measures and statements from close relatives one could have controlled better for this bias. Moreover, future studies should explore if clinical characteristics can predict outcome of reported EF 1 year later.

Nevertheless, despite these limitations, the findings in the present study add to our understanding of self-perceived cognitive difficulties in EF 1 year after initial episode in First Episode MDD patients. One strength of the present study is the uniqueness of measuring self-reported EF 1 year after initial episode and investigating the associations to depressive symptom severity and relapse the previous year. To our knowledge, no other study has conducted such a sampling. In addition, dividing the patients as regards being in remission or not fortified the study methodology, especially because the N was low and thus more vulnerable to extreme scores. This approach thus strengthens the finding that residual cognitive symptoms are present even in remission.

An additional strength of the current study is the use of a standardized, validated and reliable questionnaire of EF in everyday life. Even though BRIEF-A is increasingly used in clinical settings, to the best of our knowledge this is the first empirical report using a control group in MDD in general and in first-episode MDD in remission in particular.

### Clinical Implications

The present findings showing that former depressed patients experience neurocognitive difficulties in EF relative to healthy control subjects, somewhat independent of depressive symptom load, have several clinical implications. The results show that there is an association between symptom severity and difficulties in EF reported. However, importantly, despite being remitted, difficulties with EF in everyday life is reported 1 year after initial episode. Struggling with EF, such as initiating, planning and organizing everyday life, having problems with working memory, shifting of tasks and inhibiting negative thoughts along with problems with emotional and behavioral regulation, will certainly have a major impact in numerous arenas and settings. One can imagine that family relations, social life and work capacity and/or ability to study or go to school will be more challenging and thus affected in a negative way. Moreover, not being able to meet the domestic or work-related expectations from others or to achieve and function according to one's own expectations will potentially lead to a feeling of not being able to cope with everyday life. This could have a negative impact on self-esteem and thus potentially increase the risk of relapse.

### Targeted Interventions

The importance of identifying subjective experiences of poor everyday EF in remission, and its association to symptom severity and tendency to relapse, is most valuable in the treatment and follow up of first episode MDD patients. The knowledge regarding residual cognitive symptoms should guide clinical evaluations and decisions made by healthcare personnel and caregivers. In addition to traditional treatment that primarily targets the emotional aspects of MDD, we should target the cognitive aspects that manifest as residual symptoms in MDD. Using BRIEF-A as primary measure, Hagen et al. (54) showed improvement in self-reported EF depressive symptoms after cognitive remediation treatment. Cognitive remediation treatment is not the same as cognitive behavioral therapy, which primarily targets cognitive thoughts. This treatment instead targets cognitive functioning and aims to enhance cognitive capacity in everyday life (8). Cognitive Enhancement Therapy (CET) (8) seeks to improve cognitive and functional recovery and is composed of three main components: (1) psychoeducation, (3) practice, and (4) translation to everyday life. CET is still in its infancy, and the patient group urgently needs us to identify, acknowledge and more individually treat the residual cognitive symptoms that apparently have such a major impact on everyday life and represent a major risk for relapse. Self-report measures need to be included when screening the neuropsychological profile of the patient, in order to tailor the intervention individually.

## Conclusion

The present study showed that first-episode patients experienced significant lower EF in everyday life compared to the control group 1 year after onset. The lower EF were evident in both patients in remission and in patients with depressive symptoms, with most aspects of EF being associated depressive symptom load, both initially and 1 year after the first episode. The findings therefore indicate that EF difficulties may be affected by symptom severity, but also that such difficulties may be present despite being in remission. Further, the residual cognitive symptoms of regulatory control of behavior and emotional responses was associated with relapse. Poor EF should therefore be targeted in future interventions in order to enhance our understanding of these difficulties, increase daily life functioning and prevent relapses and/or new episodes.

## Data Availability Statement

The raw data supporting the conclusions of this article will be made available by the authors, without undue reservation.

## Ethics Statement

The studies involving human participants were reviewed and approved by Regional Committees for Medical and Health Research Ethics (REK) in Norway. The patients/participants provided their written informed consent to participate in this study.

## Author Contributions

MS collected the data and wrote the draft of the article. All authors participated in the analysis and interpretation of data, in writing the article, and approved the submitted version.

## Conflict of Interest

The authors declare that the research was conducted in the absence of any commercial or financial relationships that could be construed as a potential conflict of interest.
